# 3EZ,20Ac-ingenol, a catalytic inhibitor of topoisomerases, downregulates p-Akt and induces DSBs and apoptosis of DT40 cells

**DOI:** 10.1007/s12272-013-0108-4

**Published:** 2013-04-18

**Authors:** Yasuaki Fukuda, Masahiro Kanbe, Manami Watanabe, Katsuaki Dan, Keiichi Matsuzaki, Susumu Kitanaka, Shohei Miyata

**Affiliations:** 1 Department of Chemistry, College of Humanities and Sciences, Nihon University, Tokyo, 156-8550 Japan; 2 Collaborative Research Resources, Core Instrumentation Facility, School of Medicine, Keio University, Tokyo, 160-8582 Japan; 3 School of Pharmacy, Nihon University, Chiba, 274-8555 Japan

**Keywords:** Both topoisomerase I and II inhibitor, Catalytic inhibitor, Apoptosis, Double-strand breaks, Caspase 3, p-Akt

## Abstract

We have previously reported that many ingenol compounds derived from *Euphorbia kansui* exhibit topoisomerase (topo) II inhibitory activity. Of these compounds, 3EZ,20Ac-ingenol inhibited topo I activity. Camptothecin, which inhibits the religation activity of topo I without interfering with the binding of topo I to DNA and induces topo I-mediated DNA cleavage, was used as a positive control. In this study, we found that 3EZ,20Ac-ingenol did not hamper the binding of topo I to DNA in the same manner as camptothecin but affected the inhibition of cleavage of one DNA strand. 3EZ,20Ac-ingenol inhibited cell proliferation by blocking cell cycle progression in the G2/M phase. To define the mechanism of inhibition of DT40 cell proliferation, the change in Akt activity was observed because Akt activity is regulated in response to DNA damage. Western blot analysis revealed that 3EZ,20Ac-ingenol downregulated the expression of p-Akt, and apoptosis was detected by the presence of DNA double-strand breaks and caspase 3 activation.

## Introduction

In previously studies, we isolated ingenol compounds from *Euphorbia kansui* that inhibited topoisomerase (topo) activity and/or the proliferative activity of cancer cells (Wang et al. 2002; Miyata et al. 2006). DNA topo I and II relax helical supercoiling generated during transcription, replication, and chromatin remodeling (Wang 2002). Topo I transiently cleaves a single strand of DNA, whereas topo II cleaves double-stranded DNA (Burden and Osheroff 1998; Pommier 2006). The anti-cancer drugs camptothecin (CPT) and etoposide belong to the family of topo I and topo II inhibitors, respectively. The mechanisms of the catalytic cycle of topo I has been described as a controlled rotation process as follows: (a) topo I binds to the DNA substrate to form a topo I–DNA noncovalent complex; (b) topo I catalyzes the cleavage of one DNA strand to form a transient topo I cleavable complex; (c) controlled rotation releases the superhelical tension of DNA; (d) the cleaved DNA strand is religated; and (e) topo I is released from the relaxed DNA and undergoes another cycle of DNA relaxation (Champoux 2001). DNA topo I and II can be inhibited through different mechanisms by two classes of agents: class I (poisons) and class II (catalytic inhibitors) (Burden and Osheroff 1998; Andoh and Ishida 1998; Bailly 2003; Capranico et al. 2010; Wu et al. 2010). Class I inhibitors stabilize the DNA cleavable complex and block the subsequent rejoining of DNA breaks. When advancing replication forks collide with the drug-stabilized topo I–DNA cleavable complexes, DNA double strand breaks (DSBs) are formed (Pommier 2006). In a subsequent reaction, these DSBs induce a DNA damage checkpoint response through ATM/ATR activation and subsequent H2AX phosphorylation (Burden and Osheroff 1998; Cliby et al. 2002; Furuta et al. 2003; Pommier et al. 2006). Class II catalytic inhibitors act by inhibiting any other step of the topo-I and II enzymatic cycle and induce a decatenation checkpoint response by ATR activation (Deming et al. 2001) and subsequent G2/M arrest (Deming et al. 2001; Wu et al. 2010).

**Fig. 1:**
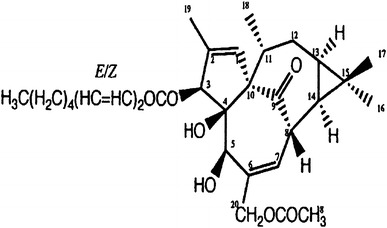
Structure of the diterpene compound, 3-*O*-(2′E,4′Z-decadienoyl)-20-*O*-acetylingenol (3EZ,20Ac-ingenol)

Within the set of topo II inhibitors investigated (Miyata et al. 2006) we found inhibitory activity of topo I in vitro by 3EZ20Ac-ingenol (Fig. 1). The present work describes experiments designed to identify mechanisms of inhibition of 3EZ,20Ac-ingenol against topo I. CPT and water-soluble derivatives of CPT are presently the most potent and poisonous (class I) topo I inhibitors. To determine whether the mode of inhibition of topo I activity by 3EZ,20Ac-ingenol is similar to that by the CPT analogue, 10-hydroxycamptothecin (hCPT), we analyzed the ability of 3EZ,20Ac-ingenol to introduce single-strand DNA breaks using plasmid DNA. In contrast to hCPT, 3EZ,20Ac-ingenol could not generate cleavable complexes, inhibit the endonuclease activity of topo I, and display characteristics of catalytic inhibitors (class II). Although, the topo I poison drugs CPT and topotecan and the topo II poison drugs adriamycin and etoposide stabilize the covalent topo–DNA cleavable complexes, thereby inducing DSBs, the topo I catalytic inhibitorβ-lapachone, the topo II catalytic inhibitor ICRF-193 and a dual catalytic inhibitor of topo I and II F 11782 do not induce DSBs (Burden and Osheroff 1998; Andoh and Ishida 1998; Capranico et al. 2010). However, we reported that although 20-*O*-ingenolEZ inhibits the ATPase activity of topo II and induces G2 arrest by inhibiting DNA decatenation in mouse mammary tumor (MMT) cells, it induces DSBs and apoptosis in BLM^−/−^ DT40 cells (Watanabe et al. 2011). Recent studies have suggested that catalytic topo II inhibitor ICRF-193 induces DSBs by acting as topo II poisons (Park and Avraham 2006; Robinson et al. 2007). In this study, using wild-type and repair-related gene-muted DT40 cells, we analyzed whether the dual catalytic inhibitor of topo 1 and II 3EZ,20Ac-ingenol from *Euphorbia* (Fig. 1) induces DNA DSBs. Phosphorylated H2AX (γH2AX), a DNA damage marker that can be used as a clinical marker of the efficacy of cancer drugs (Antony et al. 2007; Teicher 2008), was detectable at pharmacologically relevant levels in DT40 cells treated with 3EZ,20Ac-ingenol. We found that although 3EZ,20Ac-ingenol did not stabilize topo I–DNA-cleavable complexes, it induced downregulation of Akt, DSBs and apoptosis in DT40 cells.

## Materials and methods

### Cell lines and cell proliferation

BLM^−/−^ and WRN^−/−^ DT40 cells and wild-type DT40 cells were provided by the RIKEN BRC through the National Bio-Resource Project of the MEXT, Japan. The diterpene compound, 3-*O*-(2′E,4′Z-decadienoyl)-20-*O*-acetylingenol (3EZ,20Ac-ingenol) (Fig. 1) was dissolved in dimethyl sulfoxide. BLM^−/−^ and WRN^−/−^ DT40 cells and DT40 cells were incubated in RPMI 1640 supplemented for 24 h at 37 °C as described previously (Watanabe et al. 2011). Cell growth was determined by the 3-(4,5-dimethylthiazol-2-yl)-2-5-diphenyl-tetrazolium bromide (MTT) assay using the Cell Proliferation Kit I (Roche Applied Science). The optical density of each well was measured at 620 nm using a plate reader (Amersham).

### Cell cycle analysis

DT40 cells were treated with 0.5 μM 3EZ,20Ac-ingenol for 0, 6, 12, 24 and 48 h or were treated with 0.01, 0.1 and 1 μM 3EZ,20Ac-ingenol for 24 h. The cells were then harvested and fixed with 70 % ethanol for 24 h. After centrifugation at 3,000 rpm for 5 min, the cell pellets were washed with phosphate-buffered saline (PBS) and resuspended in PBS containing propidium iodide (50 μg/ml) and DNase-free RNase (50 μg/ml). The cells were then incubated at 37 °C for 20 min, and the DNA content was determined by flow cytometry (Beckman Coulter).

### pBR322 DNA plasmid relaxation assay

The effects of the compounds on relaxation by DNA topo I (human recombinant in *E. coli* TopGEN) were determined by measuring the conversion of supercoiled pBR 322 plasmid DNA to its relaxed form. The reaction mixture contained 20 mM Tris–HCl (pH 7.9), 100 mM KCl, 10 mM MgCl_2_, 0.1 mM EDTA, 50 μg/ml BSA, 100 ng pBR 322 DNA, 0.16 U of enzyme, and different concentrations of the drugs in a total volume of 20 μl. After incubation for 15 min at 37 °C, the mixtures were subjected to electrophoresis on a 1 % agarose gel. After electrophoresis, the gels were stained with ethidium bromide and photographed under UV light.

### pBR322 plasmid DNA cleavage assay

Topo I-mediated DNA cleavage assays were performed as described previously (Raspaglio et al. 2005). Plasmid DNA (10 ng) was incubated in a solution (20 μl) containing 20 mM Tris–HCl (pH 7.9), 10 mM MgCl_2_, 0.1 mM KCl, 0.1 mM EDTA, 50 μg/ml BSA, 2 U of topo I, and drugs. Reactions were initiated by the addition of DNA and incubated at 100 °C for 3 min. After digestion with proteinase K, open circular and linear DNA were separated from intact supercoiled and relaxed DNA by agarose gel electrophoresis in the presence of 1 μg/ml ethidium bromide. After electrophoresis at 4 °C, the gels were photographed under UV light.

### Analysis of topo I/DNA binding by electrophoretic mobility shift assay (EMSA)

EMSAs were performed as described previously (Boege et al. 1996). In total, 7.2 U of topo I protein was added to the reaction mixture containing 150 ng of plasmid DNA and 500 μM of 3EZ,20Ac-ingenol or 40 μM of hCPT. The assays were performed in the reaction mixture (10 mM Tris–HCl (pH7.9), 10 mM MgCl_2_, 10 mM KCl, 0.1 mM DTT, and 10 % dimethyl sulfoxide). The reaction products were immediately analyzed by electrophoresis on a 1 % agarose gel.

### 3EZ,20Ac-ingenol–DNA intercalation

The reaction buffer containing 20 mM Tris–HCl (pH 7.9), 100 mM KCl, 10 mM MgCl_2_, 0.1 mM EDTA, 50 μg/ml BSA, 150 μg of plasmid pBR322 DNA and 0.16 U of topo1 (Topo GEN) was preincubated at 37 °C for 15 min (Pommier et al. 1985). Assays were performed in the reaction buffer in the presence of 25 μM 3EZ,20Ac-ingenol or 25 μM adriamycin at 37 °C for 60 min. The reactions were halted by the addition of stop solution and electrophoresed on 1 % agarose gel.

### Immunoblotting

DT40 cells were cultured for various time periods in the presence of 0.5 μM 3EZ,20Ac-ingenol and washed in PBS. The cells were solubilized using cytoplasmic extraction reagents (Thermo Scientific). The protein concentrations were determined using the Bradford reagent for protein assays (Bio-Rad Laboratories), and 30 μg of the cell lysates was resolved on 15 % SDS polyacrylamide gels and transferred onto a polyvinylidene difluoride membrane. The blots were made using anti-p-Akt (Ser473) (Cell Signaling Technology), anti-γH2AX (Millipore), and anti-caspase 3 (R&D Systems) antibodies followed by detection using an enhanced chemiluminescence system.

## Results

### Effects of 3EZ,20Ac-ingenol on the proliferative activity of BLM^−/−^ and WRN^−/−^ DT40 cells and wild-type DT40 cells

**Fig. 2:**
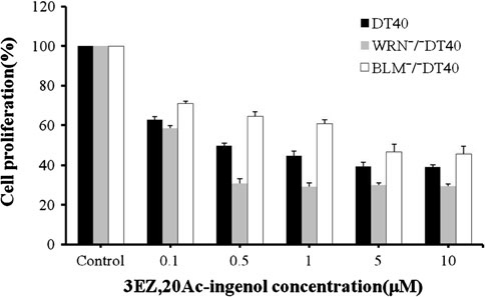
Effects of 3EZ,20Ac-ingenol on the cell proliferative activity, WRN^−/−^ and BLM^−/−^ DT40 and DT 40 cells were cultured in microplates at 37 °C for 24 h in the absence or presence of 0.1, 0.5, 1, 5 and 10 μM 3EZ,20Ac-ingenol. Relative cell growth was determined by the MTT assay. The growth of untreated WRN^−/−^ and BLM^−/−^ DT40 and wild-type DT40 cells was set as 100 %, and the growth of treated WRN^−/−^ and BLM^−/−^ DT40 and DT40 cells was expressed relative to the growth of untreated cells. The expressions were performed in triplicate, and the data are shown as the mean ± standard deviation

To investigate the inhibitory effects of 3EZ,20Ac-ingenol on the proliferation of BLM^−/−^ and WRN^−/−^ DT40 cells and wild-type DT40 cells, a concentration-response range (0.1 and 10 μM) was established using an exposure time of 24 h (Fig. 2). The proliferation of WRN^−/−^ DT40 cells and DT40 cells was gradually inhibited as the concentration of 3EZ,20Ac-ingenol increased, reaching a maximum inhibition of ~50–70 % at 0.5 μM (Fig. 2). The proliferation of WRN^−/−^ DT40 cell and wild-type DT40 cells was more sensitive to 3EZ,20Ac-ingenol than that of BLM^−/−^ DT40 cells at restricted concentrations of 0.5 and 1 μM 3EZ,20Ac-ingenol. However, there were no difference in proliferation of BLM^−/−^ cells and wild-type DT40 cells or WRN^−/−^ DT40 cell and wild-type DT40 cells at other concentrations (0.1, 5 or 10 μM). There was no difference in the inhibition of proliferation for wild-type and repair-related gene-muted DT40 cells. The proliferation of other human cancer cells such as breast cancer MCF-7 and BT-474 cells or normal B lymphocytes (HEV0011) was not inhibited by 3EZ,20Ac-ingenol at concentrations of 0.1–10 μM and the inhibition of leukemia cell (BALL-1) proliferation was more sensitive (data not shown).

### Flow cytometric analysis

**Fig. 3:**
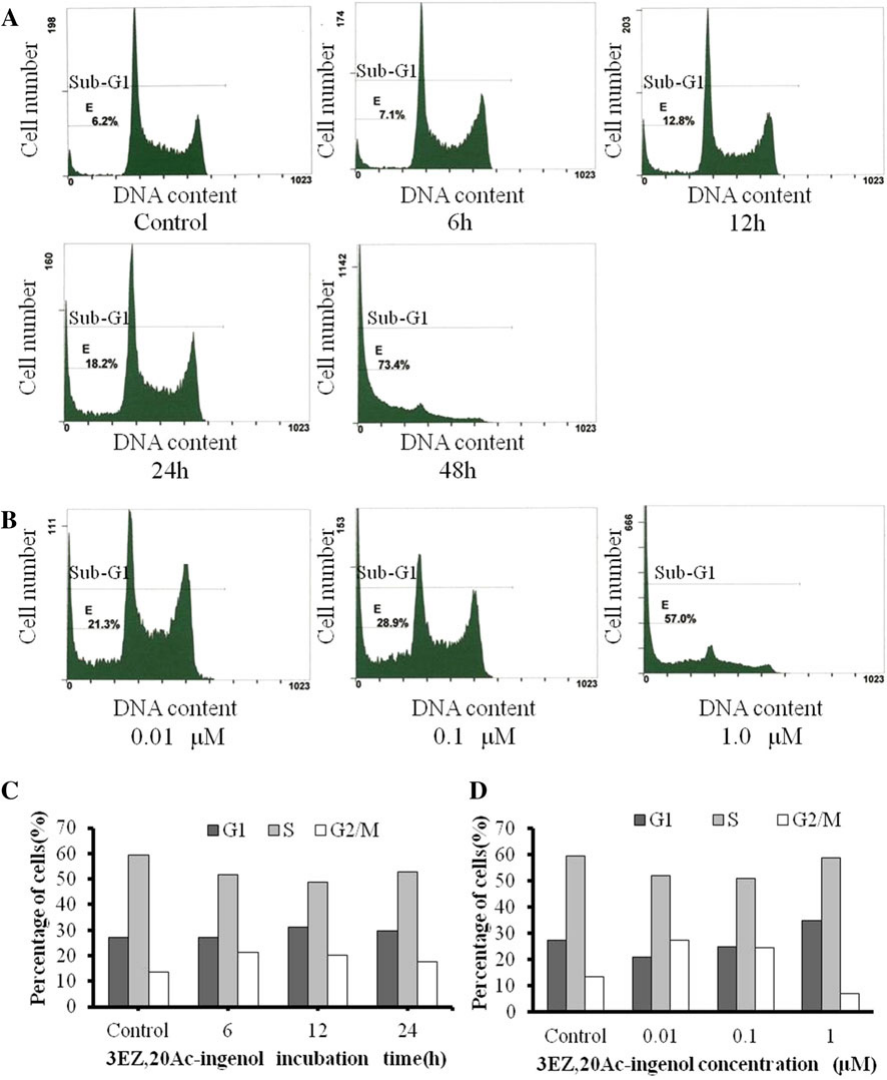
Cell cycle analysis, DT40 cells were treated with 0.5 μM 3EZ,20Ac-ingenol for 0, 6, 12, 24 and 48 h (**a**) or were treated with 0.01, 0.1 and 1 μM 3EZ,20Ac-ingenol for 24 h (**b**). The cells were stained with propidium iodide and subjected to flow-cytometric analysis. **c**, **d** shows the results of **a**, **b** with the percent distribution, respectively

We determined the effects of 3EZ,20Ac-ingenol on cell cycle distributions by flow cytometry. We treated DT40 cells with 3EZ,20Ac-ingenol at 0.5 μM for 6, 12, 24 and 48 h and then performed cell cycle analysis by flow cytometry (Fig. 3a). The results revealed that the 3EZ,20Ac-ingenol-treated DT40 cells were arrested in the G2/M phase at 6 h. At 6 h, 21 % of the cell population was in the G2/M phase, with 20 and 18 % at 12 and 24 h, respectively (Fig. 3c), compared with 14 % in the untreated cells. In contrast, in the S phase, the population was decreased to 52 and 49 % after 6 and 12 h of treatment, respectively, compared with 59 % in the untreated cells. Apoptotic progress was slow with 3EZ,20Ac-ingenol treatment until 24 h, and 18 % of cells were detected in the sub-G1 phase (Fig. 3a). This population was largely predominant at 48 h, and ~73 % of the cells were in the sub-G1 phase (Fig. 3a). The cell cycle effects of 3EZ,20Ac-ingenol were also examined as a function of the drug concentration (Fig. 3b). G2/M phase arrest occurred at a relatively low concentration (27 % at 0.01 μM as compared with 13 % in the control) and increased drug concentrations caused an obvious decline from 27 % at 0.01 μM to 24 % at 0.1 μM, 16 % at 0.5 μM, and 7 % at 1 μM after 24 of treatment (Fig. 3c, d). In contrast, DT40 cells treated with 1 μM 3EZ,20Ac-ingenol had a higher proportion, in which up to 57 % of cells had sub-G1 DNA (Fig. 3b), indicating the commitment of these cells to apoptosis. We also treated DT40 cells with 0.5 μM 3EZ,20Ac-ingenol for various periods, and the DT40 cells were most arrested in the G2/M phase after 6 h (Fig. 3c). After the G2/M phase arrest, cell apoptosis may occur selectively in G2/M phase in the presence of 3EZ,20Ac-ingenol.

### pBR322 plasmid DNA relaxation assay

**Fig. 4:**
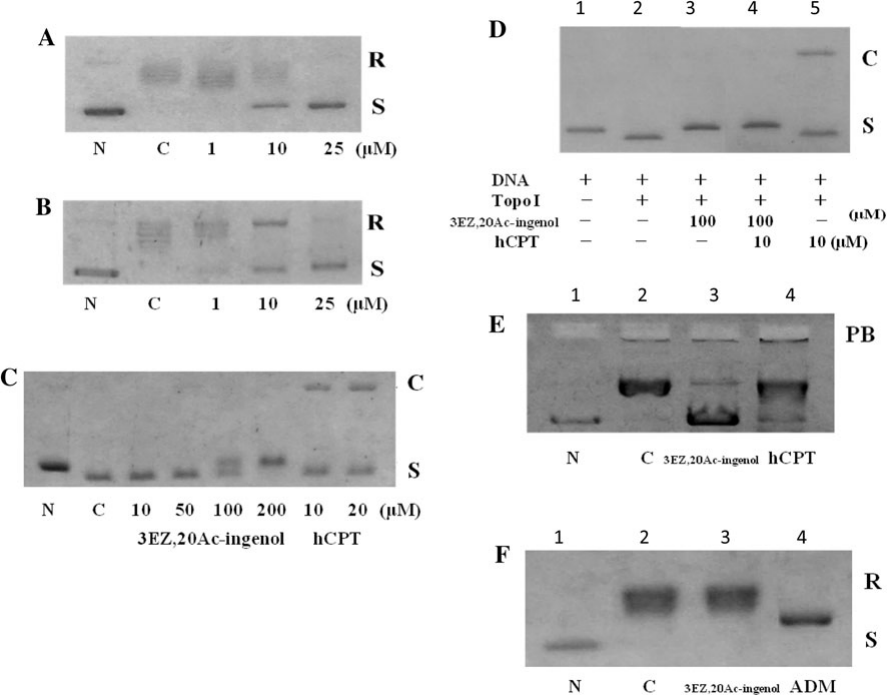
Inhibition of topo I activity by 3EZ,20Ac-ingenol and the mechanism of action. **a** Inhibition of pBR322 DNA relaxation by 3EZ,20Ac-ingenol, *N* DNA only, *C* DNA plus enzyme. **b** Inhibition of pBR322 relaxation by hCPT, *N* DNA only, *C* DNA plus enzyme. **c** The effect of 3EZ,20Ac-ingenol and hCPT on the formation of DNA-enzyme cleavable complexes, *N* DNA only, *C* DNA plus enzyme. **d** Inhibition of hCPT-induced topo I-mediated DNA cleavable complex formation by 3EZ,20Ac-ingenol. **e** Electrophoretic mobility shift assay (EMSA) to study the effect of 3EZ,20Ac-ingenol on the binding of topo I to DAN, *N* DNA only, *C* DNA plus enzyme. **f** DNA intercalation assay, *N* DNA only, *C* DNA plus enzyme. *R* relaxed DNA, *S* supercoiled DNA, *C* DNA cleavage, *PB* protein-bound DNA, *ADM* adriamycin

The topo I inhibitory activity of 3EZ,20Ac-ingenol was studied by the DNA relaxation assay. hCPT, a well-known topo I inhibitor, was used as a positive control. Inhibition of DNA relaxation by 3EZ,20Ac-ingenol was not observed at concentration less than 1 μM (Fig. 4a). 3EZ,20Ac-ingenol had slight inhibitory effects on DNA relaxation at 10 μM (Fig. 4a) and significantly inhibited DNA relaxation at 25 μM (Fig. 4a). hCPT also inhibited DNA relaxation to the same degree as 3EZ,20Ac-ingenol (Fig. 4b). We previously reported that the in vitro inhibition of topo by 3EZ,20Ac-ingenol was caused by topo II catalytic inhibition (Miyata et al. 2006). Thus, 3EZ,20Ac-ingenol inhibited topo I and II activity in vitro.

### pBR322 plasmid DNA cleavage assay

Topo I poisons such as CPT inhibit religase by affecting the cleavage step. CPT entraps a migration complex known as the cleavable complex, which is formed by the enzyme, drug, and DNA. Thus, we tested whether 3EZ,20Ac-ingenol also induced the formation of cleavable complexes similar to hCPT-like compounds. This complex can be converted irreversibly into topo-linked DNA single-strand breaks after the addition of a strong protein denaturant (Raspaglio et al. 2005). As shown in Fig. 4c, the induction of topo I-mediated DNA cleavage was detected when the concentration of hCPT was 10 or 20 μM. In contrast to hCPT, 3EZ,20Ac-ingenol had no effect on topo I-mediated DNA cleavage even at concentration as high as 200 μM (Fig. 4c).

### Inhibition of hCPT-induced DNA cleavage by 3EZ,20Ac-ingenol

Because 3EZ,20Ac-ingenol had no effect on the formation of cleavable complexes, the results indicated that the inhibition of topo I by 3EZ,20Ac-ingenol occurs upstream of the endonuclease activity of topo I and downstream of religation. Because hCPT inhibits the religation of cleaved DNA, if the interaction between DNA and topo I or the cleavage of one DNA strand to form the transient topo I cleavable complex before religation is blocked by 3EZ,20Ac-ingenol, the inhibition of hCPT-induced DNA cleavage by 3EZ,20Ac-ingenol would possible occur (Li et al. 1993). Addition of 3EZ,20Ac-ingenol to the reaction buffer affected the cleavage of DNA by hCPT (Fig. 4d). Pretreatment with 3EZ,20Ac-ingenol at 100 μM abrogated the formation of this DNA cleavable complex by hCPT, which can be explained by inhibition of the interaction between DNA and topo I or cleavage of one DNA strand in the presence of 3EZ,20Ac-ingenol (Fig. 4d, lane 4). DNA was cleaved by topo I in the presence of hCPT (Fig. 4d, lane 5).

### Binding analysis by EMSA

We showed that 3EZ,20Ac-ingenol pretreatment inhibited the formation of the cleavable complex stabilized by hCPT. Thus, we used the mobility shift assay to test whether 3EZ,20Ac-ingenol inhibited enzyme binding to DNA, which assembles a noncovalent enzyme–DNA complex, (Li et al. 1993; Raspaglio et al. 2005). The results shown in Fig. 4e, PB (lanes 2 and 3 compared with lane 1) illustrate the mobility of DNA in the presence of topo I, which is the result of a complex formed between the enzyme and DNA. Gel retardation was observed after the addition of 3EZ,20Ac-ingenol, indicating that 3EZ,20Ac-ingenol does not block the assembly of the noncovalent enzyme–DNA complex (Fig. 4e, PB, lanes 2 and 3). hCPT did not modulate the formation of the topo I–DNA complex (Fig. 4e, PB, lane 4). These results showed that 3EZ,20Ac-ingenol appears to inhibit the cleavage of one DNA strand in an earlier event of topo I-mediated DNA cleavable complex formation.

### 3EZ,20Ac-ingenol intercalation with DNA

Many chemical compounds are known to affect the gross structure of DNA by intercalation and/or binding, which can affect the catalytic activity of topo I. We tested the ability of 3EZ,20Ac-ingenol to intercalate with DNA using a topo I-catalyzed unwinding assay based on the ability of intercalating compounds to unwind the DNA duplex and change the DNA twist (Raspaglio et al. 2005). Supercoiling of the relaxed substrate DNA was induced in the presence of an intercalative drug such as adriamycin (Fig. 4f, lane 4). In contrast, supercoiling was not observed with 3EZ,20Ac-ingenol (Fig. 4f, lane 3).

### Influence of 3EZ,20Ac-ingenol on the PI3K/Akt pathway in DT40 cells

**Fig. 5:**
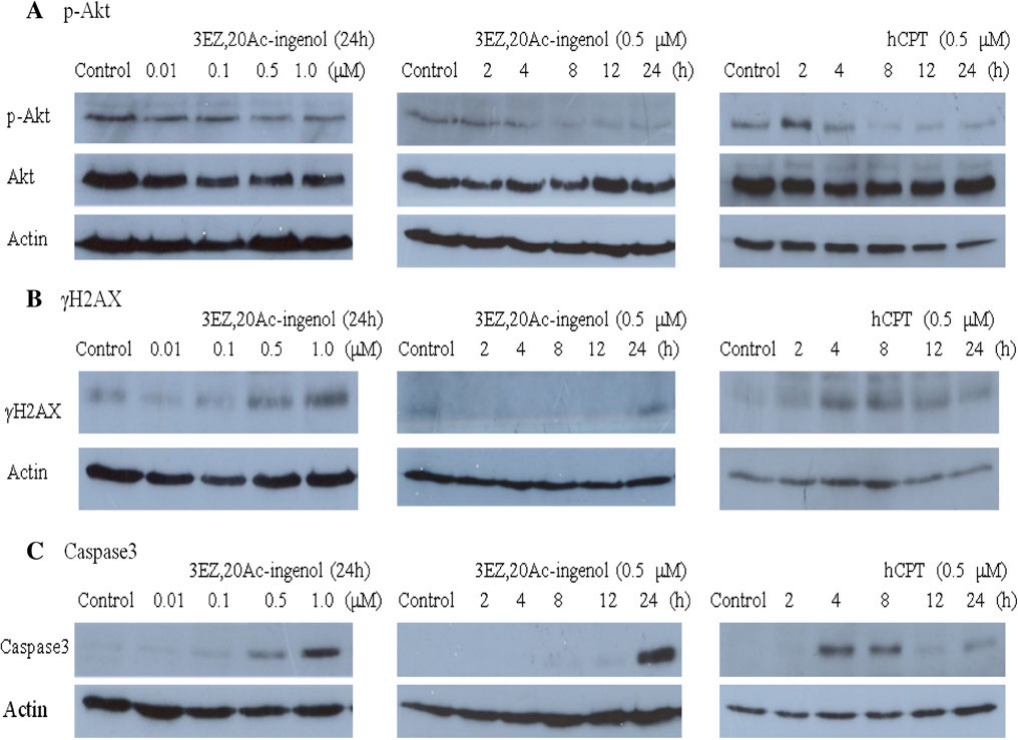
Analysis of p-Akt content, phosphorylated H2AX (γH2AX) formation, and caspase 3 activation by immunoblotting. **a** Influence of 3EZ,20Ac-ingenol on p-Akt protein expression. **b** Influence of 3EZ,20Ac-ingenol on H2AX phosphorylation. **c** Influence of 3EZ,20Ac-ingenol on caspase 3 activation. **a**–**c** (*left*) DT40 cells were treated with 0.01, 0.1, 0.5 and 1 μM 3EZ,20Ac-ingenol for 24 h; (*center*) DT40 cells were treated with 0.5 μM 3EZ,20Ac-ingenol for 2, 4, 8, 12, and 24 h; and (*right*) DT40 cells were treated with 0.5 μM hCPT for 2, 4, 8, 12, and 24 h

PI3K/Akt activity has been demonstrated to be regulated by genotoxins, and it plays important roles in cell apoptosis (Roos and Kaina 2012; Xu et al. 2012). The inhibition of p-Akt was observed in DT40 cells treated with 0.5 μM 3EZ,20Ac-ingenol, 8 h after 3EZ,20Ac-ingenol treatment, whereas the expression level of total Akt protein experienced little or no change (Fig. 5a). The inhibition of p-Akt in hCPT-treated cells was also observed after 4–8 h, which was almost simultaneous with the downregulation of p-Akt in 3EZ,20Ac-ingenol treated DT40 cells (Fig. 5a).

### Influence of 3EZ,20Ac-ingenol on H2AX phosphorylation in DT40 cells

γH2AX which serves as a marker of DNA DSBs, was visualized as a band stained with anti-γH2AX in DT 40 cells treated with 0.5 μM of 3EZ,20Ac-ingenol (Fig. 5b). Time-course analysis revealed H2AX phosphorylation after 12–24 h of 3EZ,20Ac-ingenol treatment. γH2AX was observed in hCPT treated cells after 2–4 h, revealing the extremely early appearance of γH2AX compared with H2AX phosphorylation in 3EZ,20Ac-ingenol treated cells (Fig. 5b).

### Influence of 3EZ,20Ac-ingenol on caspase 3 activation

3EZ,20Ac-ingenol-treatment activated caspase 3 in DT40 cells in a concentration-dependent manner. Caspase 3 activation was observed in response to 0.5 μM of 3EZ,20Ac-ingenol and after 24 h at the same concentration (Fig. 5c). In contrast to the 3EZ,20Ac-ingenol treated cells, caspase 3 activation in hCPT-treated cells occurred after 4 h (Fig. 5c), which was slightly late than theγH2AX formation in hCPT treated cells (Fig. 5b).

## Discussion

In this study, we observed that 3EZ,20Ac-ingenol interacted with topo I–DNA complexes and inhibited topo I-mediated DNA cleavage. It was previously shown 3EZ,20Ac-ingenol was a topo II catalytic inhibitor (Miyata et al. 2006). Therefore, 3EZ,20Ac-ingenol inhibits both topo I and II activity in vitro. The catalytic topo inhibitor, 3EZ,20Ac-ingenol induced H2AX phosphorylation in response to agents that induce the formation of topo I-mediated DSBs in DT40 cells. Apoptosis was detected in 3EZ,20Ac-ingenol treated cells by γH2AX accumulation and caspase 3 activation during 12–24 h of treatment and through cell cycle analysis by flow cytometry between 24 and 48 h. To define the mechanisms of apoptosis induction, we studied whether 3EZ,20Ac-ingenol downregulated p-Akt (Ser 473) expression. We revealed that downregulation of p-Akt was initiated in DT40 cells after 8 h of treatment with 3EZ,20Ac-ingenol.

The formation of topo I-induced DNA DSBs can occur through two distinct mechanisms (Sordet et al. 2004). The first type is topo I poison. These include clinically used drugs, for example, the prototypical topo I poison, CPT, which stabilizes topo I–DNA cleavable complexes and induces the accumulation of cleaved DNA, resulting in chromosomal damage (Pommier et al. 2006). ATM/ATR is activated by DSBs formation and mediates subsequent H2AX phosphorylation and activation of checkpoint kinase, thereby inducing apoptosis via p53 (Cliby et al. 2002; Furuta et al. 2003; Roos and Kaina 2006). The formation of DNA DSBs by topo I poisons can be detected by γH2AX during the initial 1 h of treatment (Furuta et al. 2003). We also observed the appearance of γH2AX after 2 h of hCPT treatment.

Topo I and II poisons are commonly used as chemotherapeutic agents. However, topo I catalytic inhibitors that exhibit anti-tumor effects have also identified in cell culture (Yanagihara et al. 2005; Bentle et al. 2006; Ganguly et al. 2007; Capranico et al. 2010; Wu et al. 2010). The second type topo induced DSBs are not formed directly and are related to endogenously produced reactive oxygen species (ROS) (Bentle et al. 2006; Ganguly et al. 2007; Kluza et al. 2006). For example, betulinic acid (Ganguly et al. 2007) binds with topo I, thereby precluding its interaction with substrate DNA (Chowdhury et al. 2002). Events such as ROS generation and DNA fragmentation were observed in betulinic acid treated cells after 2–4 h, and ROS-induced apoptotic processes are the key steps in the blockade of cell cycle progression. β-lapachone (Li et al. 1993; Bentle et al. 2006) interacts with topo I rather than the DNA substrate and blocks the formation of cleavable complexes, however, it dose not induce the topo1-mediated DNA cleavage. β-lapachone induces the generation of NQO1-dependent ROS, DNA breaks, and γH2AX formation in the treated cells. γH2AX induced by ROS endogenously produced in response to the β-lapachone occurred during the initial 30–90 min of treatment (Bentle et al. 2006).

It has been reported that ICRF-193, a catalytic topo II inhibitor, induces DSBs and apoptosis through activation of checkpoint kinas (Park and Avraham 2006; Robinson et al. 2007) or traps topo II into a circular clamp on DNA, which arrests transcription and triggers proteasomal degradation of topo II (Xiao et al. 2003). However, the mechanism of induction of DSBs and apoptosis by topo I catalytic inhibitors remains unclear (Bentle et al. 2006; Kluza et al. 2006; Ganguly et al. 2007; Capranico et al. 2010). The mechanism for inducing DSBs and apoptosis of DT40 cells treated with the catalytic inhibitor 3EZ,20Ac-ingenol remains unknown. However, we consider that DNA damage derived from inhibiting DNA decatenation through the inhibition of topo I mediated DNA cleavage by 3EZ,20Ac-ingenol during the G2 phase is the key lesion. We previously reported the effects of the catalytic topo II inhibitor 20-*O*-ingenolEZ on the cell cycle distributions of MMT cells by flow cytometry (Yoshida et al. 2010). At 12 h, 16.5 % of the cell population was in the G2/M phase. After 24 and 48 h of treatment, these values were 30 and 27 %, respectively, as compared with 12 % in untreated cells. MMT cells treated with 20-*O*-ingenolEZ were arrested in G2/M phase, but were not induced in apoptosis, whereas DT40 cells treated with the inhibiter were induced to undergo apoptosis (Yoshida et al. 2010; Watanabe et al. 2011). In this study, DT40 cells treated with the dual catalytic inhibitor of topo I and II 3EZ,20Ac-ingenol were also selectively arrested in G2/M phase and were induced to undergo apoptosis through signaling by cell cycle checkpoints in G2/M phase. Because DT40 cell lines do not express p53 (Takao et al. 1999), they are arrested in G2/M phase by catalytic topo I and/or II inhibitory activity of 3EZ,20Ac-ingenol and may be induced to undergo apoptosis through p53-independent decatenation checkpoint pathway (Deming et al. 2001; Roos and Kaina 2006).

Genotoxins inhibit the Akt pathway in response to DNA damage, and they overcome the suppression of apoptosis by Akt to activate apoptosis effectively (Roos and Kaina 2012; Xu et al. 2012). The downregulation of p-Akt (Ser 473) is observed between 12 and 24 h of treatment by topotecan or SN-38 (Nakashio et al. 2000; Liu et al. 2009). The downregulation of p-Akt in betulinic acid-treated cells was also observed between 12 and 24 h of treatment and occurred after the ROS generation and DNA fragmentation (Ganguly et al. 2007; Zhao et al. 2012). Apoptosis is gradually induced at early time points, as indicated by DNA damage, and apoptotic evolution is strongly induced after 36 and 48 h of treatment, as observed by the downregulation of p-Akt (Nakashio et al. 2000; Liu et al. 2009; Zhao et al. 2012). We showed that the downregulation of Akt in DT40 cells treated with hCPT was observed after 4 h and was almost the simultaneous with the appearance of DSBs and activation of caspase 3 in the treated DT40 cells. However, we demonstrated that the downregulation of p-Akt occurred in DT40 cells treated with 3EZ,20Ac-ingenol, and this downregulation was followed by the induction of H2AX phosphorylation and caspase 3 activation and subsequently followed by the intense induction of apoptosis through transition of cells to the sub-G1 phase between 24 and 48 h. Because p53 is frequently mutated in tumors (Hollstein et al. 1999) and increased Akt kinase activity is observed in some tumor cell lines (Xu et al. 2012), more studies on 3EZ,20Ac-ingenol, which induces decatenation checkpoint through G2/M arrest by catalytic topo inhibitor and downregulates p-Akt, may provide useful information for developing anti-cancer agents.

## References

[CR1] Andoh T, Ishida R (1998). Catalytic inhibitors of DNA topoisomerase II. Biochimica et Biophysica Acta.

[CR2] Antony S, Agama KK, Miao ZH, Takagi K, Wright MH, Robles AI, Varticovski L, Nagarajan M, Morrell A, Cushman M, Pommier Y (2007). Novel indenoisoquinolines NSC 725776 and NSC 724998 produce persistent topoisomerase I cleavage complexes and overcome multidrug resistance. Cancer Research.

[CR3] Bailly C (2003). Homocamptothecins: Potent topoisomerase I inhibitors and promising anticancer drugs. Critical Reviews in Oncology Hematology.

[CR4] Bentle MS, Reinicke KE, Bey EA, Spitz DR, Boothman DA (2006). Calcium-dependent modulation of poly(ADP-ribose) polymerase-1 alters cellular metabolism and DNA repair. Journal of Biological Chemistry.

[CR5] Boege F, Straub T, Kehr A, Boesenberg C, Christiansen K, Andersen A, Jakob F, Köhrle J (1996). Selected novel flavones inhibit the DNA binding or the DNA religation step of eukaryotic topoisomerase I. Journal of Biological Chemistry.

[CR6] Burden DA, Osheroff N (1998). Mechanism of action of eukaryotic topoisomerase II and drugs targeted to the enzyme. Biochimica et Biophysica Acta.

[CR7] Capranico G, Marinello J, Baranello L (2010). Dissecting the transcriptional functions of human DNA topoisomerase I by selective inhibitors: implications for physiological and therapeutic modulation of enzyme activity. Biochimica et Biophysica Acta.

[CR8] Champoux JJ (2001). DNA topoisomerases: Structure, function, and mechanism. Annual Review of Biochemistry.

[CR9] Chowdhury AR, Mandal S, Mittra B, Sharma S, Mukhopadhyay S, Majumder HK (2002). Betulinic acid, a potent inhibitor of eukaryotic topoisomerase I: Identification of the inhibitory step, the major functional group responsible and development of more potent derivatives. Medical Science Monitor.

[CR10] Cliby WA, Lewis KA, Lilly KK, Kaufmann SH (2002). S phase and G2 arrests induced by topoisomerase I poisons are dependent on ATR kinase function. Journal of Biological Chemistry.

[CR11] Deming PB, Cistulli CA, Zhao H, Graves PR, Piwnica-Worms H, Paules RS, Downes CS, Kaufmann WK (2001). The human decatenation checkpoint. Proceedings of the National Academy of Sciences of the United States of America.

[CR12] Furuta T, Takemura H, Liao ZY, Aune GJ, Redon C, Sedelnikova OA, Pilch DR, Rogakou EP, Celeste A, Chen HT, Nussenzweig A, Aladjem MI, Bonner WM, Pommier Y (2003). Phosphorylation of histone H2AX and activation of Mre11, Rad50, and Nbs1 in response to replication-dependent DNA double-strand breaks induced by mammalian DNA topoisomerase I cleavage complexes. Journal of Biological Chemistry.

[CR13] Ganguly A, Das B, Roy A, Sen N, Dasgupta SB, Mukhopadhayay S, Majumder HK (2007). Betulinic acid, a catalytic inhibitor of topoisomerase I, inhibits reactive oxygen species-mediated apoptotic topoisomerase I–DNA cleavable complex formation in prostate cancer cells but does not affect the process of cell death. Cancer Research.

[CR14] Hollstein M, Hergenhahn M, Yang Q, Bartsch H, Wang ZQ, Hainaut P (1999). New approaches to understanding p53 gene tumor mutation spectra. Mutation Research.

[CR15] Kluza J, Mazinghien R, Irwin H, Hartley JA, Bailly C (2006). Relationships between DNA strand breakage and apoptotic progression upon treatment of HL-60 leukemia cells with tafluposide or etoposide. Anti-Cancer Drugs.

[CR16] Li CJ, Averboukh L, Pardee AB (1993). beta-Lapachone, a novel DNA topoisomerase I inhibitor with a mode of action different from camptothecin. Journal of Biological Chemistry.

[CR17] Liu Y, Xing H, Weng D, Song X, Qin X, Xia X, Weng Y, Liang F, Chen G, Han X, Ma X, Wang S, Zhou J, Xu G, Meng L, Ma D (2009). Inhibition of Akt signaling by SN-38 induces apoptosis in cervical cancer. Cancer Letters.

[CR18] Miyata S, Wang LY, Yoshida C, Kitanaka S (2006). Inhibition of cellular proliferation by diterpenes, topoisomerase II inhibitor. Bioorganic & Medicinal Chemistry.

[CR19] Nakashio A, Fujita N, Rokudai S, Sato S, Tsuruo T (2000). Prevention of phosphatidylinositol 3′-kinase-Akt survival signaling pathway during topotecan-induced apoptosis. Cancer Research.

[CR20] Park I, Avraham HK (2006). Cell cycle-dependent DNA damage signaling induced by ICRF-193 involves ATM, ATR, CHK2, and BRCA1. Experimental Cell Research.

[CR21] Pommier Y (2006). Topoisomerase I inhibitors: Camptothecins and beyond. Nature Reviews Cancer.

[CR22] Pommier Y, Barcelo JM, Rao VA, Sordet O, Jobson AG, Thibaut L, Miao ZH, Seiler JA, Zhang H, Marchand C, Agama K, Nitiss JL, Redon C (2006). Repair of topoisomerase I-mediated DNA damage. Progress in Nucleic Acid Research and Molecular Biology.

[CR23] Pommier Y, Minford JK, Schwartz RE, Zwelling LA, Kohn KW (1985). Effects of the DNA intercalators 4′-(9-acridinylamino) methanesulfon-m-anisidide and 2-methyl-9-hydroxyellipticinium on topoisomerase II mediated DNA strand cleavage and strand passage. Biochemistry.

[CR24] Raspaglio G, Ferlini C, Mozzetti S, Prislei S, Gallo D, Das N, Scambia G (2005). Thiocolchicine dimers: a novel class of topoisomerase-I inhibitors. Biochemical Pharmacology.

[CR25] Robinson HM, Bratlie-Thoresen S, Brown R, Gillespie DA (2007). Chk1 is required for G2/M checkpoint response induced by the catalytic topoisomerase II inhibitor ICRF-193. Cell Cycle.

[CR26] Roos WP, Kaina B (2006). DNA damage-induced cell death by apoptosis. Trends in Molecular Medicine.

[CR27] Roos, W.P., and B. Kaina. 2013. DNA damage-induced apoptosis: From specific DNA lesions to the DNA damage response and apoptosis. *Cancer Letters* 332: 237–248. 10.1016/j.canlet.2012.01.007 22261329

[CR28] Sordet O, Khan QA, Pommier Y (2004). Apoptotic topoisomerase I–DNA complexes induced by oxygen radicals and mitochondrial dysfunction. Cell Cycle.

[CR29] Takao N, Kato H, Mori R, Morrison C, Sonada E, Sun X, Shimizu H, Yoshioka K, Takeda S, Yamamoto K (1999). Disruption of ATM in p53-null cells causes multiple functional abnormalities in cellular response to ionizing radiation. Oncogene.

[CR30] Teicher BA (2008). Next generation topoisomerase I inhibitors: Rationale and biomarker strategies. Biochemical Pharmacology.

[CR31] Wang JC (2002). Cellular roles of DNA topoisomerases: A molecular perspective. Nature Reviews Molecular Cell Biology.

[CR32] Wang LY, Wang NL, Yao XS, Miyata S, Kitanaka S (2002). Diterpenes from the roots of *Euphorbia kansui* and their in vitro effects on the cell division of Xenopus. Journal of Natural Products.

[CR33] Watanabe M, Kamada Y, Miyazaki K, Mizoguchi S, Matsuzaki K, Kitanaka S, Miyata S (2011). 20-*O*-ingenolEZ, a catalytic topoisomerase II inhibitor, specifically inhibits cell proliferation and induces double-strand DNA breaks in BLM^−/−^ cells. Medicinal Chemistry Communications.

[CR34] Wu N, Wu XW, Agama K, Pommier Y, Du J, Li D, Gu LQ, Huang ZS, An LK (2010). A novel DNA topoisomerase I inhibitor with different mechanism from camptothecin induces G2/M phase cell cycle arrest to K562 cells. Biochemistry.

[CR35] Xiao H, Mao Y, Desai SD, Zhou N, Ting CY, Hwang J, Liu LF (2003). The topoisomerase IIbeta circular clamp arrests transcription and signals a 26S proteasome pathway. Proceedings of the National Academy of Sciences of the United States of America.

[CR36] Xu N, Lao Y, Zhang Y, Gillespie DA (2012). Akt: A double-edged sword in cell proliferation and genome stability. Journal of Oncology.

[CR37] Yanagihara M, Sasaki-Takahashi N, Sugahara T, Yamamoto S, Shinomi M, Yamashita I, Hayashida M, Yamanoha B, Numata A, Yamori T, Andoh T (2005). Leptosins isolated from marine fungus *Leptoshaeria* species inhibit DNA topoisomerases I and/or II and induce apoptosis by inactivation of Akt/protein kinase B. Cancer Science.

[CR38] Yoshida C, Hishiyama K, Miyazaki K, Watanabe M, Kanbe M, Yamada Y, Matsuzaki K, Miyashita K, Kitanaka S, Miyata S (2010). Analysis of inhibition of topoisomerase II alpha and cancer cell proliferation by ingenolEZ. Cancer Science.

[CR39] Zhao Z, Wang J, Tang J, Liu X, Zhong Q, Wang F, Hu W, Yuan Z, Nie C, Wei Y (2012). JNK- and Akt-mediated Puma expression in the apoptosis of cisplatin-resistant ovarian cancer cells. Biochemical Journal.

